# Effect of erythropoietin on primed leucocyte expression profile

**DOI:** 10.1098/rsob.140026

**Published:** 2014-06-11

**Authors:** Mirko Pesce, Paolo Felaco, Sara Franceschelli, Lorenza Speranza, Alfredo Grilli, Maria Anna De Lutiis, Alessio Ferrone, Vittorio Sirolli, Mario Bonomini, Mario Felaco, Antonia Patruno

**Affiliations:** 1 Department of Psychological, Humanistic and Territorial Sciences, University ‘G. D'Annunzio’, Chieti, Italy; 2 Department of Medicine and Science of Aging, University ‘G. D'Annunzio’, Chieti, Italy

**Keywords:** peripheral blood mononuclear cells, tumour necrosis factor alpha, erythropoietin-*α*, immune mediators, MAPK p38, Erk1/2

## Abstract

Resistance to erythropoietin (EPO) affects a significant number of anaemic patients with end-stage renal disease. Previous reports suggest that inflammation is one of the major independent predictors of EPO resistance, and the effects of EPO treatment on inflammatory mediators are not well established. The aim of this study was to investigate EPO-induced modification to gene expression in primary cultured leucocytes. Microarray experiments were performed on primed *ex vivo* peripheral blood mononuclear cells (PBMCs) and treated with human EPO-α. Data suggested that EPO-α modulated genes involved in cell movement and interaction in primed PBMCs. Of note, EPO-α exerts anti-inflammatory effects inhibiting the expression of pro-inflammatory cytokine IL-8 and its receptor CXCR2; by contrast, EPO-α increases expression of genes relating to promotion of inflammation encoding for IL-1β and CCL8, and induces *de novo* synthesis of IL-1α, CXCL1 and CXCL5 in primed cells. The reduction in MAPK p38-α activity is involved in modulating both IL-1β and IL-8 expression. Unlike the induction of MAPK, Erk1/2 activity leads to upregulation of IL-1β, but does not affect IL-8 expression and release. Furthermore, EPO-α treatment of primed cells induces the activation of caspase-1 upstream higher secretion of IL-1β, and this process is not dependent on caspase-8 activation. In conclusion, our findings highlight new potential molecules involved in EPO resistance and confirm the anti-inflammatory role for EPO, but also suggest a plausible *in vivo* scenario in which the positive correlation found between EPO resistance and elevated levels of some pro-inflammatory mediators is due to treatment with EPO itself.

## 2. Introduction

Anaemia is an inevitable condition that develops early in the course of chronic kidney disease (CKD) and increases in frequency while the glomerular filtration rate further declines [1]. With the advent of erythropoiesis-stimulating agents (ESAs) in the 1990s, great improvements were achieved in the treatment of anaemia in the majority of patients with end-stage renal disease, but 10% of all treated patients remained anaemic despite large ESA doses; those patients are therefore considered resistant to ESA treatment [2,3]. A number of factors have been implicated as contributors to ESA resistance, though persistent inflammation with higher levels of pro-inflammatory cytokines is increasingly being appreciated as the major cause of EPO hyporesponsiveness [4–7].

Pro-inflammatory cytokines simultaneously affect erythropoiesis at several levels, and manifest their effects as ‘inflammation anaemia’ and hyporesponsiveness to EPO treatment in patients with CKD [8]. Pro-inflammatory cytokines can, indeed, cause EPO resistance, inhibiting the *in vitro* growth of erythroid progenitors [9–11] and interfering with iron homeostasis. In particular, tumour necrosis factor (TNF-α), IL-1 and IL-6 increase the expression of divalent metal transporter-1, induce ferritin synthesis, reduce ferroportin expression and stimulate the transferrin-bound iron reuptake by increasing the transferrin receptor expression in macrophages. TNF-α and IL-1 can also damage the erythrocyte membranes and stimulate erythrocyte phagocytosis [12,13]. All these processes promote the intracellular accumulation of iron by reducing its bioavailability, reduce the production of endogenous EPO as well as the bone marrow response to the erythropoietic stimulus, and end by making and keeping patients anaemic [14].

EPO is a multi-functional cytokine, which exerts erythropoietic and immune-modulatory effects upon binding to two distinct receptors, which are expressed on erythroid, parenchymal and immune cells [15]. The bond between EPO-α and EPO receptor (EPOR) in peripheral blood mononuclear cells (PBMCs) leads to a reduction in inflammation [16,17]. However, EPO administration was also found to further augment the lipopolysaccharide (LPS)-induced increase in plasma levels of TNF-α and IL-6, and to attenuate the decrease in lymphocyte number [18]. Again, it has been reported that anaemia in CKD is associated with upregulation of TNF-α, along with increased levels of this pro-inflammatory cytokine in patients treated with ESAs [19]. These findings suggest that the effects of EPO treatment on inflammatory mediators are strictly dependent on the phase and intensity of the inflammatory condition present during drug administration.

Hence, inflammation markers may be considered sensitive outcome predictors in patients suffering from CKD and thus involved in EPO resistance. Gene expression being a sensitive endpoint, a microarray can be useful in monitoring the exposure effect and identifying any new biomarkers. Accordingly, in this study, we applied gene expression microarray technology to identify the differentially expressed genes in PBMCs, primed or not, when exposed to human recombinant erythropoietin-α (EPO-α). We chose PBMCs because they are the main source of inflammation mediators, present EPOR on their membrane, and have been defined as surrogate markers and key mediators in low-grade inflammation associated with renal failure. These cells may exist in the bloodstream in a quiescent, primed or activated state. The ‘primed’ state involves PBMCs first encountering a stimulus [20]. Keeping in mind that patients who are hyporesponsive to ESAs show high baseline serum levels of TNF-α correlated with a negative response to ESAs, PBMCs primed cells were obtained by exposure to this pro-inflammatory cytokine [21].

## 3. Material and methods

### 3.1. Chemicals

The following were used: TNF-α (Enzo Life Science, Lousen, Switzerland); LPS (*Escherichia coli* 0127:B), MAPK p38 inhibitor SB203580, Erk1/2 inhibitor PD98059, Caspase-1 inhibitor Ac-YVAD-cmk, Caspase-8 inhibitor Z-IETD-FMK (Sigma, St. Louis, MO); recombinant human EPO-α (RayBiotech, Norcross, GA).

### 3.2. Cell culture

Buffy coats were collected from the blood of 10 healthy individual donors from the Transfusion Blood Bank Services of Chieti, Italy. All donors gave informed consent and no individuals were taking medication. PBMCs were isolated as previously described [22]. The cell viability, determined by trypan blue exclusion, was more than 99%. The same batch of serum and medium was used in all experiments. The media and sera were characterized by very low LPS content as determined by testing the chromogenic assay using limulus amoebocyte lysate (minimum detection level 0.1 μg ml^−1^; Whittaker Bioproducts, Walkersville, MD). PBMCs obtained from each subject were prepared following the same experimental procedure and divided into four samples for microarray experiment: (i) control cells, (ii) cells stimulated with TNF-α (10 ng ml^−1^, 30 min), (iii) cells stimulated with EPO-α (20 U ml^−1^, 15 min), and (iv) cells pre-stimulated with TNF-α (10 ng ml^−1^, 15 min) and then treated with EPO-α (20 U ml^−1^, 15 min). EPO-α was used at a concentration of 20 U ml^−1^, which has previously been shown significantly to attenuate polymorphonuclear leucocyte (PMNL) priming and PMNL-associated systemic low-grade inflammation [23]. The inhibitor of MAPK p38 (SB203580) or Erk1/2 (PD98059) was used at a concentration of 1 μM and added 30 min prior to TNF-α stimulation; antibody anti-EPO (MAB287, R&D Systems, Minneapolis, MN) was used at a concentration of 10 μg ml^−1^ and added 30 min prior to EPO-α stimulation [24]. LPS was used at a concentration of 10 μg ml^−1^ [22]; Caspase-1 inhibitor Ac-YVAD-cmk or Caspase-8 inhibitor Z-IETD-FMK was used at a concentration of 10 and 20 μM, respectively, and added 1 h prior EPO-α stimulation [25,26]. Cells were seeded onto six-well tissue culture plates and incubated overnight at 37°C in a humidified atmosphere of 5% CO_2_.

### 3.3. Microarray

Total RNA extraction from human PBMCs was performed using Trizol reagent (Invitrogen, Carlsbad, CA). The RNA concentration and purity were determined by measuring absorbencies at 260 and 280 nm, a 260 : 280 ratio of 1.9 being considered acceptable for analysis. The extracted RNA was first run on a denaturing gel to make sure that two bright distinct bands, representing the 28S and 18S ribosomal species, were found. This confirmed that the RNA lacked DNA contamination and was not degraded, both of which might confound the array results. Analysis was carried out using a high-density array containing about 30 968 human genome probes and 1082 experimental control probes (60-mer sense-strand polynucleotide probes; Eurogentec, Seraing, Belgium) [27]. Briefly, 100 ng of RNA of each buffy coat (*n* = 10) were pooled for all four samples and then amplified using the Ammino Allyl MessageAmp II aRNA Amplification Kit (Ambion, Austin, TX), which is able to produce aRNA, containing 5-(3-amminoallyl)-UTP-modified nucleotides. The aRNA (5–20 μg) obtained was labelled with Cys3 or Cys5 fluorochromes (Amersham, Pharmacia Biotech, Buckinghamshire, UK) and hybridized on the array. aRNA obtained from control cells (1) was labelled with Cys3 (Cys5 in dye swap experiments). aRNA obtained from samples—cells stimulated with TNF-α (2), cells treated with EPO-α (3) and cells treated with EPO-α after stimulation with TNF-α (4)—was labelled with Cys5 (Cys3 in dye swap experiments). Each experiment was performed using different microarray slides, in which labelled aRNA from control cells (1) was hybridized with labelled aRNA from sample 2 or 3 or 4. Fluorescent signals were captured by ScanArray 5000 Packard laser scanning (Packard BioChip Technologies, Billerica, MA) and normalized using ScanArray Express software. Microarray data are deposited in the GEO public database (accession no.: GSE53336). All data are MIAME compliant.

### 3.4. Real time PCR

Total RNA was extracted in the same way as for microarray. RNA (1 μg) from each buffy coat (*n* = 10) for all four samples was treated with DNAse I (Fermentas) and cDNA was obtained using the ‘high-capacity cDNA’ kit (Applied Biosystems), according to the manufacturer's instructions.

A qPCR assay was carried out in an Eppendorf Mastercycler EP Realplex (Eppendorf AG). Preliminary qPCR reactions were run to optimize the concentration and ratio of each primer set. For all the cDNA templates, 2 μl was used in a 20 μl qPCR amplification system provided by the SYBR Green Real Master Mix Kit, according to the manufacturer's directions. Primers for human IL1A, IL1B, CCL8, CXCL1, CXCL5, IL8, CXCR2 and GAPDH as a reference gene (table 1) were designed using GeneWorks software (IntelliGenetix, Mountain View, CA).

**Table 1.** Sequences of human primers used for qPCR analysis.

species	gene name	reference sequence	primers
*Homo sapiens*	GADPH	NM_002046	For GCGCCCAATACGACCAA Rev GCTCTCTGCTCCTCCTGTT
	IL1A	NM_000575.3	For TCCCAATCTCCATTCCCAAAC Rev CTCTACCAAGGACCAGAGAGAA
	IL1B	NM_000576.2	For CAAAGGCGGCCAGGATATAA Rev CTAGGGATTGAGTCCACATTCAG
	CXCL1	NM_001511.3	For GGAACAGAAGAGGAAAGAGAGAC Rev TAGGACAGTGTGCAGGTAGA
	CXCL5	NM_002994.3	For CCTGAAGAACGGGAAGGAAA Rev CTGCTGAAGACTGGGAAACT
	IL8	NM_000584.3	For CTTGGCAGCCTTCCTGATTT Rev GGGTGGAAAGGTTTGGAGTATG
	CXCR2	NM_001557.3	For CTCGTGATGCTGGTCATCTTAT Rev CAAGGTCAGGGCAAAGAGTAG
	CCL8	NM_005623.2	For TCATTGTTCTCCCTCCTACCT Rev GCACTGATTGCCAAAGAATACC

Similar amplification procedures and data computation were followed as described above. No qPCR products were generated from genomic versus cDNA template. The fluorescence intensity of double-strand-specific SYBR Green, reflecting the amount of qPCR product formed, was monitored at the end of each elongation step. Melting curve analysis was performed to confirm the purity of the qPCR products. Relative expression of IL1A, IL1B, CCL8, CXCL1, CXCL5, IL8 and CXCR2 was normalized to GAPDH using the ΔCT method (relative expression = 2^−ΔCT^, where ΔCT = CT (IL1A; IL1B; CCL8; CXCL1; CXCL5; IL8; CXCR2) − CT(GADPH)). Quantification cycle (Cq) values were exported directly onto Excel worksheets for analysis. Relative changes in gene expression were determined by the 2^–ΔΔCT^ method as described previously [28] and reported as the difference (*n*-fold) relative to the value for a calibrator cDNA (control) prepared in parallel with the experimental cDNAs. DNA was denatured at 95°C for 2 min followed by 40 cycles of 30 s at 95°C together with 30 s at 60°C. The experiments were repeated twice with consistent results.

### 3.5. ELISA

Quantitative measurement of human cytokines was assayed using specific ELISA development systems to detect IL-1β, IL-1α and IL-8 (Searchlight, Aushon Biosystems, MA) in accordance with the manufacturer's instructions.

### 3.6. Western blotting

Western blotting was performed as described below [29]. Briefly, total protein extracts were prepared by treating cells with lysis buffer (RIPA). In Western blot analysis, 50 μg of protein per lane was separated on a 4–12% NuPAGE gradient gel (Gibco Invitrogen, Paisley, UK). Blots were probed and incubated with rabbit polyclonal IgG anti-p38, anti-p-p38 (Thr 180/Tyr 182), anti-Erk1/2, anti-p-Erk1/2 (Thr202/Tyr204), anti-CXCL1, anti-CXCL5 and anti-CCL8 (Santa Cruz Biotechnology, Santa Cruz, CA) at 0.2 μg ml^−1^ in Tris-buffered saline 0.1% Tween-20. A mouse anti-human monoclonal antibody recognizing the human β-actin (A5441; Sigma-Aldrich) was used as control in all experiments. Immunoblot signals were developed using Super Signal Ultra chemiluminescence detection reagents (Pierce Biotechnology, Rockford, IL). The blot images were analysed with a gel analysis software package (Gel Doc 1000; Bio-Rad).

### 3.7. Statistical analysis

Data were analysed using paired *t*-test statistics with SPSS 18.0.1 (SPSS, Chicago, IL) for Windows. Results are described as mean ± s.d. for each assessment performed at least in triplicate. The level of statistically significant difference was defined as *p* < 0.05. p38α and p-p38α, and Erk1/2 and p-Erk1/2 levels at each time point were compared by non-parametric Mann–Whitney *U*-test with Bonferroni correction.

The microarray-obtained data were statistically analysed using the SAM system (where SAM is significance analysis of microarray). Genes were median centred and SAM multiclass analysis was performed. Missing values were calculated using the K-nearest-neighbour algorithm set to 100. A false discovery rate = 0% was used for multiclass analysis.

Ingenuity Pathway Analysis (IPA, v. 8.8) software (Mountain View, CA) was used to identify the association between the uploaded genes with the biological functions and canonical pathways present in the Ingenuity Pathways Knowledge Base. The Benjamini–Hochberg multiple testing correction was applied to determine the level of significance of biological functions (*p* < 0.001). Fisher's exact test was applied to determine the significance of pathway associations (*p* < 0.01). A *Z*-score of more than or less than 2 was accepted for prediction analysis.

## 4. Results

### 4.1. Microarray analysis

SAM multiclass analysis was performed among the gene expression variations obtained from three classes: control cells versus cells primed with TNF-α, control cells versus cells treated with EPO-α and control cells versus cells treated with both stimuli. The analysis generated a significantly perturbed list of 124 genes (FDR = 0%) that was uploaded onto IPA (Ingenuity Systems, Redwood City, CA) for assignment of biological functions as well as to identify any perturbed signal transduction pathways. Electronic supplementary material, file S1 shows the complete gene list analysed. The results showed that the biological functions modulated by treatment with each stimulus alone or in combination involved key functions of immune system regulation. The most significant are listed in table 2 (B–H, *p* < 0.001). The alteration of gene expression is mainly implicated in regulation of cellular development, though also in PBMC dynamic processes, such as cell movement and cell-to-cell interaction.

**Table 2.** Functions significantly modulated in the gene list.

function	B–H *p*-value	molecules
cellular movement	5.71 × 10^−7^–6.84 × 10^−2^	CIB1,IL8,ID2,IL1A,FN1,THBS1,EGR1,DGKA,CD36, S100A4,CXCL1,CXCL5,CLIC4,CTSZ,CLEC11A,CD9, CXCR2,PECAM1,CCL8,CD151
hair and skin development and function	5.71 × 10^−7^–6.84 × 10^−2^	IL8,ID2,FN1,CXCR2,THBS1,CD36,CXCL1,CXCL5
cardiovascular system development and function	2.32 × 10^−6^–6.84 × 10^−2^	IL8,IL1A,FN1,THBS1,DGKA,CXCL1,CD36,CXCL5,CLIC4, LPAR6,CD9,CXCR2,PECAM1,CD151
haematological system development and function	4.22 × 10^−6^–6.84 × 10^−2^	MRC1,IL8,IL1A,TNFRSF9,FN1,FES,THBS1,CXCL1,CD36, CXCL5,LILRB1,CTSZ,CLEC11A,CD9,CXCR2,CEBPD,PECAM1, CCL8,CD151,MNDA
immune cell trafficking	4.22 × 10^−6^–6.84 × 10^−2^	MRC1,IL8,IL1A,FN1,FES,THBS1,CXCL1,CXCL5,LILRB1, CTSZ,CXCR2,PECAM1,CCL8,CD151
cell-to-cell signalling and interaction	1 × 10^−4^–6.84 × 10^−2^	MRC1,CIB1,IL8,IL1A,FN1,FES,THBS1,EGR1,CXCL1,CD36, CXCL5,LILRB1,CD9,CXCR2,PECAM1,MAP1LC3A,CD151
tissue development	1 × 10^−4^–6.08 × 10^−2^	CIB1,MRC1,IL8,IL1A,FN1,FES,THBS1,EGR1,DGKA,CXCL1, CD36,CD9,CXCR2,PITPNM1,PECAM1,CD151
cell morphology	1.36 × 10^−4^–6.84 × 10^−2^	CIB1,IL8,TNFRSF9,IL1A,FN1,FES,THBS1,CXCL1,CLIC4, AMD1,CTSZ,CEP170,LPAR6,SCN1B,CD9,CXCR2,PITPNM1, PECAM1,MAP1LC3A,CCL8,CASP8,CD151
connective tissue development and function	2.24 × 10^−4^–6.08 × 10^−2^	IL8,IL1A,FN1,THBS1,CXCR2,CD36,TNFAIP3
skeletal and muscular system development and function	5.35 × 10^−4^–6.44 × 10^−2^	IL8,ID2,IL1A,FN1,THBS1,S100A4,TNFAIP3,CLIC4

The pathway analysis was performed upon cytokine signalling. The analysis revealed significant differential expression for genes involved in IL-1 signalling (*p* < 0.01). Thus, IPA predicted significant activation for IL-1α in inducing upregulation of itself and other pro-inflammatory mediators in PBMCs treated with both stimuli (*Z*-score of more than 2). These genes encode for chemokines CXCL1, CXCL5 and CCL8. Table 3 shows the values of fold change (FC) for the above-mentioned genes and for the β isoform of IL-1. Variations of the expression reported for the β isoform of IL-1 showed induction upon stimulation with TNF-α, and induction of higher intensity when cells were treated with both stimuli. However, the high variability between replicates obtained did not allow this cytokine to be included in the list of significant genes, and called for further quantitative assessment which will be described below.

**Table 3.** Expression variations for key genes involved in inflammation obtained in microarray analysis (n.s., not significant).

	TNF-α	EPO-α	TNF-α + EPO-α
	FC (mean ± s.d.)	FC (mean ± s.d.)	FC (mean ± s.d.)
IL1A	n.s.	n.s.	5.68 ± 1.35^a^
IL1B	2.86 ± 2.53	n.s.	7.87 ± 6.08
IL8	3.19 ± 1.27^a^	n.s.	n.s.
CXCL1	n.s.	n.s.	3.19 ± 0.40^a^
CXCL5	n.s.	n.s.	3.34 ± 0.55^a^
CCL8	2.26 ± 1.19^a^	n.s.	6.12 ± 1.65^a^
CXCR2	n.s.	n.s.	−4.54 ± 1.72^a^
FES	n.s.	n.s.	−1.71 ± 0.16^a^

^a^Significant genes considering the FDR = 0%.

The anti-inflammatory action of EPO-α transpires from the normalization of the expression on the transcript of pro-inflammatory cytokine IL-8 and the downregulation of gene coding for its receptor CXCR2. Also, the gene encoding for kinase Feline Sarcoma Oncogene (FES), involved in regulating the haematopoietic process and chemotaxis, is significantly downregulated in cells treated with both stimuli.

### 4.2. Microarray validation data

In order to quantitatively evaluate the changes to gene expressions obtained by microarray, we performed qPCR experiments. Table 4 shows that the expression levels follow trends similar to those obtained in microarray. In particular, the expression of IL-1β that is significantly induced by TNF-α further increases upon treating primed PBMCs with EPO-α.

**Table 4.** Changes in the expression of genes involved in inflammation obtained from qPCR analysis.

	IL1A	IL1B	CXCL1	CXCL5	IL8	CXCR2	CCL8
CTRL	1.00 ± 0.04	1.00 ± 0.08	1.00 ± 0.12	1.00 ± 0.14	1.00 ± 0.32	1.00 ± 0.18	1.00 ± 0.21
TNF-α	1.07 ± 0.17	2.53 ± 0.12**	1.17 ± 0.13	1.05 ± 0.25	3.45 ± 0.19**	1.09 ± 0.17	2.48 ± 0.15**
EPO-α	0.94 ± 0.10	0.97 ± 0.11	1.05 ± 0.18	1.04 ± 0.19	0.97 ± 0.12	0.96 ± 0.16	1.14 ± 0.19
TNF-α + EPO-α	3.05 ± 0.68*	6.05 ± 0.15*	2.99 ± 0.26*	3.33 ± 0.27*	1.05 ± 0.45*	0.32 ± 0.19*	4.72 ± 0.11*

*p < 0.05 versus cells treated with TNF-α, **p < 0.05 versus control PBMCs.

**Figure 1.**
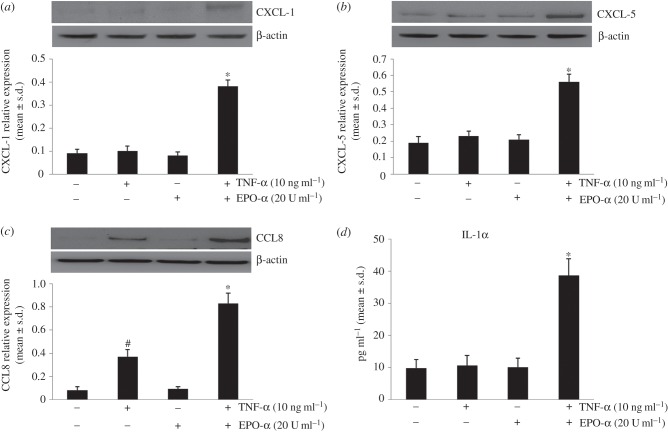
Effect of EPO-α on protein expressions of chemokines and release of IL-1α. PBMC cells were treated with TNF-α and/or EPO-α at the doses indicated. Western blot analysis of (*a*) CXCL-1, (*b*) CXCL-5 and (*c*) CCL8 expression in PBMCs. Cells were pre-treated or not with TNF-α and then treated with EPO-α. Each immunoreactive band was analysed by densitometry and normalized to β-actin levels. (*d*) ELISA measurement of IL-1α levels in conditioned medium of PBMCs. The medium levels of IL-1α increase in primed cells treated with EPO-α (*p < 0.05). Results are expressed in picogram per millilitre (mean ± s.d.). In all experiments, data shown are expressed as mean ± s.d.; *p < 0.05 compared with PBMCs primed with TNF-α; ^#^*p* < 0.05 compared with control PBMCs; data were from at least three independent experiments, each performed in triplicate (*n* = 9).

Next, we investigated by Western blotting experiments whether the modulation exerted by EPO-α on primed cells was also induced at protein level. As shown in figure 1, the CXCL1, CXCL5 and CCL8 protein levels present similar trends observed in microarray and qPCR analyses. We also observed the same trend in measuring the release of IL-1α in conditioned medium of PBMCs under the same experimental conditions (figure 1*d*). These data suggest that there did not occur any post-transcriptional regulation mechanisms during these processes.

### 4.3. Role of MAPK p38α and Erk1/2 activations on erythropoietin-α regulation of IL-8 and IL-1β expression and release in primed peripheral blood mononuclear cells

Since our data suggested some controversial effects by EPO-α on pro-inflammatory mediators, particularly on TNF-α upregulation of IL-1β and IL-8, we further investigated the mechanisms involved in this process, analysing the expression of MAPK p38α and Erk1/2, and their relative phosphorylated isoforms. The role of p38 has been previously described with regard to IL-8 expression regulation in several cell types stimulated with TNF-α [30–32]. Several reports showed that p38 could be involved in resolution of inflammation and thus in negative regulation of pro-inflammatory cytokine expression [33–36]. Furthermore, the involvement of Erk1/2 has been associated with IL-1β induction [37,38].

**Figure 2.**
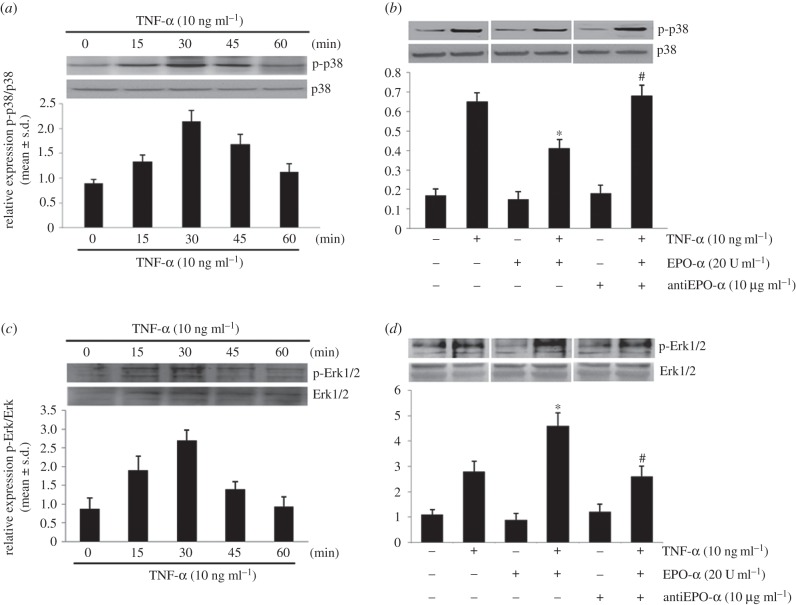
Effects of TNF-α and EPO-α treatment on MAPK p-p38 and Erk1/2 expression. The expression of (*a*) p-p38α^(Thr180/Tyr182)^ and p38α proteins, and (*c*) p-Erk1/2^(Thr202/Tyr204)^ and Erk1/2, in representative time course Western blot experiments (top). At the bottom, relative expression of (*a*) p-p38α/p38α and (*c*) p-Erk/Erk (mean ± s.d., *n* = 3) in PBMCs primed with TNF-α. Western blotting representative image of (*b*) p-p38α and p38α, and (*d*) p-Erk1/2 and Erk1/2, protein expression in PBMCs treated or not with TNF-α (10 ng ml^−1^, 30 min), and EPO-α and/or anti-EPO-α (top). Each immunoreactive band was analysed by densitometry and normalized to (*b*) p38-α or (*d*) Erk1/2 levels (mean ± s.d., *n* = 6) (bottom). *p < 0.01 versus TNF-α-treated cells, ^#^*p* < 0.01 versus TNF-α- and EPO-α-treated cells.

As shown in figure 2*a*, the treatment of PBMCs with TNF-α induced a higher level of p-p38α^(**Thr180/Tyr182)**^ protein than quiescent cells (0 min), and the peak of induction was observed at 30 min after TNF-α stimulation (*p* < 0.01). Figure 2*b* shows the effects of the EPO-α treatment on p-p38α protein expression. The treatment with EPO-α alone does not affect p-p38α levels when applied to control cells, whereas the treatment with EPO-α significantly decreases the level of phosphorylated form of p38α (*p* < 0.05). Blockage of EPO-α with a specific Ab anti-EPO significantly abrogates the EPO-α reduction of p-p38α in primed PBMCs.

In addition, the treatment of PBMCs with TNF-α induced a higher level of p-Erk1/2^(Thr202/Tyr204)^ protein, with the peak of induction observed at the same time as p-p38α (figure 2*c*). Unlike from p38, the EPO-α treatment significantly increases on the levels of p-Erk1/2 expression in primed cells (*p* < 0.05) when compared with TNF-α stimulated cells (figure 2*d*). As observed for p-p38 expression modulation, pre-treatment of PBMCs with specific Ab anti-EPO abrogates the effect on p-Erk1/2 expression.

In order to better clarify the role of EPO-α in pro-inflammatory cytokine IL-1β and IL-8 gene expression, SB203580 and PD98059 were used to treat PBMCs as a MAPK p38α and Erk1/2 selective inhibitor, respectively. qPCR experiments were performed. The results shown in table 5 suggest that inhibition of p38 activity significantly reinforces the trends observed for IL-1β and IL-8 expression in primed cells treated with EPO-α. In detail, we observed that p38 inhibition further increased IL-1β expression in primed PBMCs treated with EPO-α. Again, pre-treatment with SB203580 inhibitor significantly abrogated the expression normalization of IL-8 exerted by treating primed cells with EPO-α.

**Table 5.** Effect of p38 and Erk1/2 selective inhibitors and EPO-α blockage on IL1B and IL8 gene expression in PBMCs. mRNA expression was determined by qPCR. The relative increase in mRNA expression (mean ± s.d., *n* = 10) is shown.

	IL1B (mean ± s.d.)	IL8 (mean ± s.d.)
control	1.00 ± 0.09	1.00 ± 0.12
TNF-α	2.63 ± 0.10	3.45 ± 0.20
EPO-α	1.02 ± 0.08	0.97 ± 0.10
anti-EPO-α	1.08 ± 0.11	1.05 ± 0.12
SB203580	0.96 ± 0.07	1.01 ± 0.09
PD98059	1.09 ± 0.10	1.11 ± 0.14
TNF-α + EPO-α	6.11 ± 0.16*	1.05 ± 0.15*
TNF-α + EPO-α + SB203580	8.28 ± 0.18**	2.83 ± 0.21**
TNF-α + EPO-α + PD98059	4.63 ± 0.21**	1.06 ± 0.11
TNF-α + EPO-α + anti-EPO-α	2.81 ± 0.25**	3.25 ± 0.27**

*p < 0.05 versus PBMCs treated with TNF-α, **p < 0.05 versus PBMCs treated with TNF-α and EPO-α.

The pre-treatment of primed cells stimulated with EPO-α with PD98059 significantly reduced the mRNA amount of IL-1β, but did not affect those of IL-8. Thus, as far as MAPK Erk1/2 is concerned, our findings suggest the involvement of this kinase in mediating the EPO-α induction of IL-1β expression. Again, these expression modulations were completely blocked by Ab anti-EPO-α, suggesting that bound EPO/EPOR occurs in this process.

Of note, we did not observe significant modulation in treating cells with MAPK inhibitors alone (table 5).

The cytokine biological effects were elicited by protein product. In particular, IL-1β is expressed at the transcriptional level as a precursor form, while only the active form is cleaved and secreted. Subsequently, we performed ELISA measurements for these cytokines, comparing how they were released in the conditioned medium of primed PBMCs when compared with EPO-α.

**Figure 3.**
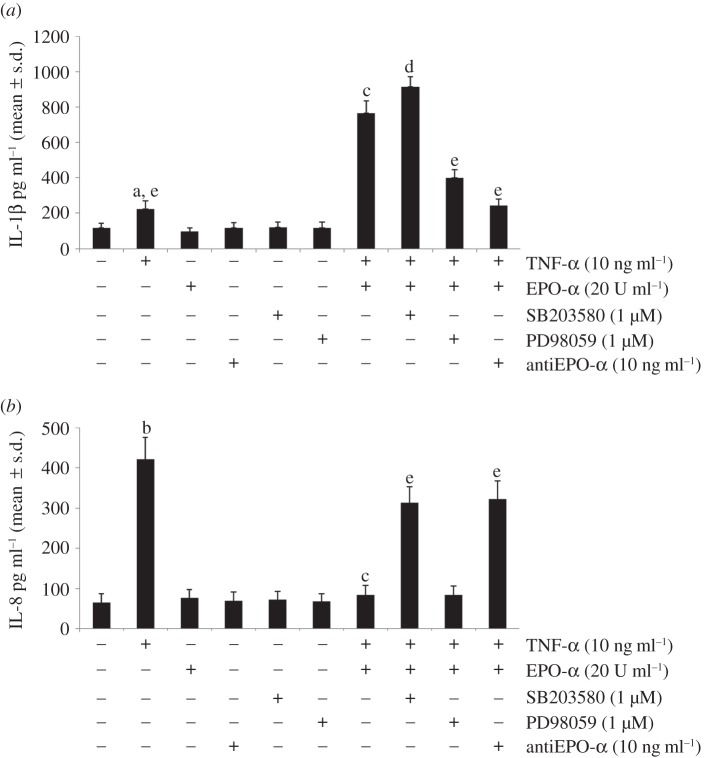
Effect of p38 and Erk1/2 selective inhibitors and EPO-α blockage on (*a*) IL-1β and (*b*) IL-8 cytokines secretion in conditioned PBMC medium. The levels of cytokines, as measured by ELISA, are given as mean ± s.d. (*n* = 6). ^a^*p* < 0.05, ^b^*p* < 0.01 versus no treated cells; ^c^*p* < 0.01 versus TNF-α-treated cells; ^d^*p* < 0.05, ^e^*p* < 0.01 versus PBMCs treated with TNF-α and EPO-α.

The release of IL-8 in conditioned medium of PBMCs showed a similar trend to that reported for the expression level (figure 3*b*). Furthermore, we observed increased secretion of IL-1β after stimulation with TNF-α and a still higher induction treating primed cells with EPO-α (figure 3*a*), suggesting the presence of a mechanism that increases the formation of the active-secreted form of IL-1β.

### 4.4. Effect of protease inhibitors on IL-1β secretion by primed peripheral blood mononuclear cells treated with erythropoietin-α

The caspases were proteases strictly classified into two subfamilies depending on whether they controlled inflammation, such as caspases-1 (CASP-1), or apoptotic cell death, such as caspases-8 (CASP-8). The CASP-1 activations are dependent on the inflammasomes and their cytosolic activation platform; CASP-1 then catalyse the proteolytic maturation of pro-inflammatory cytokines IL-1β and IL-18 [39]. However, recent findings suggested that the CASP-8 also exert non-apoptotic roles in inflammation and immunity [40].

**Figure 4.**
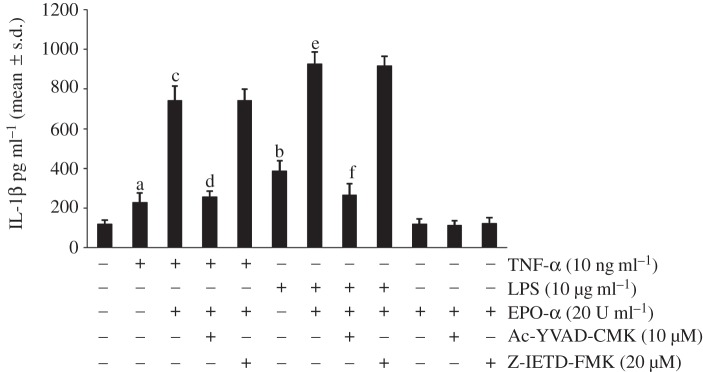
Effect of CASP-1 and CASP-8 selective inhibitors on IL1β secretion in conditioned PBMCs medium. The levels of cytokine, as measured by ELISA, are given as mean ± s.d. (*n* = 6). ^a^*p* < 0.05, ^b^*p* < 0.01 versus no treated cells; ^c^*p* < 0.01 versus TNF-α-treated cells; ^d^*p* < 0.01 versus PBMCs treated with TNF-α and EPO-α; ^e^*p* < 0.01 versus LPS-treated cells; ^f^*p* < 0.01 versus PBMCs treated with LPS and EPO-α.

In order to verify whether EPO-α acts as inflammasome activator in eliciting IL-1β secretion in primed cells, we performed ELISA experiments on conditioned medium of PBMCs. The cells were primed with TNF-α or LPS, pre-treated with CASP-1 or CASP-8 selective inhibitor and then treated or not with EPO-α. The data reported in figure 4 suggest that EPO-α treatment of cells primed with LPS showed a higher amount of released IL-1β versus cells primed with TNF-α. Of note, pre-treatment of primed PBMCs with CASP-1 selective inhibitor significantly abrogates these processes, but we did not report any variations inhibiting CASP-8 activity under the same experimental conditions.

## 5. Discussion

In past years, increasing evidence of associations between chronic inflammation and hyporesponsiveness to erythropoietic stimulation warranted investigation of the molecules characterizing the process, which might prove useful as new therapeutic targets. EPO hyporesponsiveness*/*resistance seems to be due to several interacting factors, such as an altered ratio of cytokines stimulating and inhibiting erythropoiesis as well as interference with post-receptor intracellular signalling between pro-inflammatory cytokines and ESAs. Furthermore, the effects of ESA treatment on inflammation mediators proved controversial and needed further investigation [41]. Thus, we focused our attention on expression modulation as exerted by EPO-α on primed PBMCs, and started functional analysis of the kinases involved at a post-receptor level. Analysis of the microarray data showed that 124 genes are differentially expressed through our classes of analysis (SAM analysis, FDR = 0%). These genes mainly play a role in specific immunity functions such as migration and cell–cell interactions, suggesting that, at least *in vitro*, leucocytes primed and unprimed show different responses to EPO-α treatment. We analysed these genes strictly in relation to cytokine signalling and observed that the signalling of IL-1 is significantly modulated by TNF-α/EPO-α synergy. Treatment with EPO-α further increased the TNF-α induction of gene expression and secretion of active forms of IL-1β, and stimulated *de novo* expression of IL-1α in primed cells. In particular, we observed an additive effect by EPO-α on IL-1β, which proves higher for the release of the active form in conditioned medium, when compared with the increase in the precursor form at a transcriptional level. These findings suggest that under our experimental conditions, EPO could act as a secretor stimulus on primed PBMCs. We hypothesized that this process is dependent on the interaction exerted by EPO on CASP-1, a key component of the inflammasome, which leads to IL-1β precursor activation [42], but not CASP-8 activity; this induction was indeed annulled by pre-treatment of cells with CASP-1 selective inhibitor (Ac-YVAD-AMK), but not when PBMCs were pre-incubated with selective CASP-8 inhibitor (Z-IETD-FMK). In accordance with the above fact, microarray analysis suggested that the CASP-8 gene is significantly downregulated in primed cells treated with EPO-α when compared with the other classes of analysis (electronic supplementary material, file S1). By contrast, quiescent PBMCs showed no differences in gene expression after stimulation with EPO-α.

Unlike β-isoform, both the precursor and the mature form of IL-1α are biologically active [43]. Our data showed that the expression of gene encoding for the precursor of α-isoform and the protein release is upregulated after treatment of cells with TNF-α and EPO-α. We did not observe any significant difference when treating PBMCs with either stimulus alone. We also found that EPO further increased CCL8 and induced *de novo* mRNA and protein expression of CXCL1 and CXCL5 in primed cells. IPA analysis suggested that these upregulations were significantly associated with upstream induction of IL-1α (*Z*-score > 2).

Drawing some first conclusions: we have confirmed that pro-inflammatory cytokines of the IL-1 family exert various EPO-α effects on PBMCs, whether primed or not. The cytokines IL-1α and β have already been strongly associated with establishment and maintenance of the anaemic state in CKD patients, inhibiting the *in vitro* growth of erythroid progenitors, exerting a negative interaction on iron homeostasis, damaging erythrocyte membranes, and therefore proving to be important molecules in eliciting a blunted response to EPO. Of note, the polymorphism IL1B-511-CC, associated with a variety of diseases in which inflammation plays an important role, is at present considered as a genetic marker for the EPO requirement in haemodialysis patients [44]. Again, we propose the chemokines CXCL1, CXCL5 and CCL8 be considered as potential new biomarkers for EPO resistance. These chemokines are involved in the chemoattraction of leucocytes to inflammatory sites and strongly contribute to chronic inflammation [45]. Moreover, the effect on erythroid precursor cell growth and iron metabolism has not yet been evaluated.

On the other hand, we show negative modulation in the case of important molecules involved both in the early stages and in the continuation of inflammation, confirming the anti-inflammatory effect of EPO previously described *in vivo* [16,17]. In particular, the expression and secretion of IL-8 was impaired by stimulation with TNF-α and abrogated by treatment with EPO-α. Similarly, gene coding for its receptor CXCR2 is significantly downregulated upon applying both stimuli. CXCR2 receptor can also bind other chemokines, including CXCL1 and CXCL5 [46]. Thus, downregulation of it plausibly involves a reduction of the potential pro-inflammatory effects exerted through CXCL1 and CXCL5 binding that are upregulated under the same experimental conditions. The inhibiting effect that EPO-α exerts on IL-8 expression *in vitro* could be significant *in vivo*. IL-8 is expressed mainly by monocytes and macrophages in the early stages of inflammation and persists for an extended period. This cytokine is responsible for the recruitment of neutrophils to the site of inflammation and subsequent activation of them [47]. What is more, through its angiogenic properties, IL-8 induces migration and proliferation of endothelial cells and smooth muscle cells, contributing to plaque formation in atherosclerosis [48]. It is reasonable to assume that this cytokine participates in the complexes that involve inflammation, malnutrition and atherosclerosis, and that portend a poor prognosis in CKD patients [49].

Nowadays, the only cytokine that is yet associated with ESA resistance is IL-6; it is in fact considered a strong predictor of ESA hyporesponsiveness in haemodialysis patients who have sufficient iron [50]. Among the cytokines and chemokines described in our work, none of them has been yet proposed as an ESA resistance predictor. Indeed, no study to date has investigated their levels in patients' ESA-hyporesponsiveness versus control. However, some studies on rodents confirmed the trends reported in our results concerning the two most relevant pro-inflammatory cytokines involved in ESA resistance (i.e. IL-1β and IL-8). Wu *et al.* [51] showed that a dose of 300 U kg^−1^ intravenous administration of EPO increases serum level of IL-1β. What is more, rats used in their experiments were sepsis-induced by LPS prior to EPO administration [51]. Furthermore, in another study it was proposed that a higher dose (1000 U kg^−1^) of EPO intravenous administration reduced the serum level of IL-8 after induction of ischaemic/reperfusion process [52].

Altogether, our results showed that the effects of EPO-α on inflammatory mediator expression are elaborate and in some respects controversial. To further clarify the mechanisms that underlie these processes, we started by considering the post-receptor level, investigating the expression of MAPKs p38 and Erk1/2. In particular, p38 is reportedly involved in EPO signalling and in IL-1β and IL-8 expression modulation [32,33,37,38,53,54].

We observed that the normalization exerted by EPO-α treatment on IL-8 transcript expression and protein secretion levels in primed cells involves a reduction in MAPK p38 activity, shown by reduction of the phosphorylated levels of the alpha isoform, but not those of Erk1/2. These findings may be explained by previous studies, which observed that the very low amount of IL-8 found in quiescent cells is a result of very unstable mRNA, and that activation of the MAPK p38 pathway markedly stabilizes IL-8 mRNA [53]. Of note, more recently, Dauletbaev *et al.* [55] suggested that the downregulation of TNF-induced IL-8 requires the inhibition of p38 via MAPK phosphatase 1-dependent and -independent mechanisms.

In addition, we show here that these forms of expression modulation were strongly blocked by Ab anti-EPO, indicating that EPO/EPOR binding is necessary in this process.

Our results also clearly demonstrate that the reduction in MAPK p38 activity induced by EPO-α in primed cells plays a part in strengthening IL-1β expression. The molecular mechanism by which this occurs might be explained by EPO-α reducing the role of p38 in anti-inflammatory processes [34–36]. In this connection, we noted that potent and selective inhibition of MAPK p38 significantly increases IL-1β mRNA expression and secretion in primed cells treated with EPO-α. Blockage of EPO-α with selective antibody completely inhibits the process.

Furthermore, we showed that EPO-α induces higher levels of MAPK Erk1/2 activity in primed cells. Interestingly, the pre-treatment of primed PBMCs treated with EPO-α by the selective inhibitor PD98059 significantly abrogates IL-1β secretion in conditioned medium. On the other hand, we did not report significant differences for IL-8 under the same experimental conditions.

Finally, we reported the downregulation exerted by EPO-α on genes encoding for FES in primed PBMCs. This kinase and FER protein (FES-related) are the only two members of a subfamily of proteins characterized by cytoplasmatic tyrosine kinase activity [56]. FES has been associated with growth factors and receptors for cytokines, which increase its catalytic activity by phosphorylation of tyrosine sites [57,58]. A role of FES in compartmentalization of myeloid and innate immunity has been highlighted through the study of mouse model knock-out or with a truncated form of this protein [56,59,60]. Of note, recent data on human erythroleukemia cells show that, following receptor stimulation upstream of FES, this kinase undergoes transient phosphorylation, which is essential for cell chemotaxis [61].

In summary, our study reinforces the hypothesis that inflammation has a key role in EPO hyporesponsiveness/resistance. We were the first to describe the interesting modulation in gene expression of chemical mediators of inflammation. These genes are very likely to be involved in EPO resistance mechanisms and contribute to enriching the previously reported effects of EPO on immune cells. We have demonstrated that modulation of MAPK p38 and Erk1/2 activities seem to be involved in IL-1β expression regulation and activation of CASP-1 in secretion of this cytokine. Furthermore, reduction of p38 activity seems to be involved in EPO-α regulation of IL-8. The exact mechanisms of EPO-α treatment on primed cells remain to be clarified. To this end, we hope in future to further integrate our expression data with functional analysis of other kinases. We also intend to measure *in vivo* the molecular targets we have highlighted, in order to throw light on any additional markers in patients hyporesponsive to EPO.

## Supplementary Material

Supplementary file 1
